# Body position and cuff size influence lower limb arterial occlusion pressure and its predictors: implications for standardizing the pressure applied in training with blood flow restriction

**DOI:** 10.3389/fphys.2024.1446963

**Published:** 2024-08-12

**Authors:** Victor S. de Queiros, Nicholas Rolnick, Okan Kamiş, Magno F. Formiga, Roberto F. C. Rocha, Júlio César Medeiros Alves, João Guilherme Vieira, Jeferson Macedo Vianna, Michal Wilk, Krzysztof Fostiak, Breno Guilherme de Araújo Tinôco Cabral, Paulo Moreira Silva Dantas

**Affiliations:** ^1^ Graduate Program in Health Sciences, Federal University of Rio Grande do Norte (UFRN), Natal, Brazil; ^2^ Department of Exercise Science and Recreation, CUNY Lehman College, New York, NY, United States; ^3^ The Human Performance Mechanic, New York, NY, United States; ^4^ Department of Sports and Health, Aksaray University, Aksaray, Türkiye; ^5^ Graduate Program in Physiotherapy and Functioning, Department of Physiotherapy, Federal University of Ceará, Fortaleza, Brazil; ^6^ Graduate Program in Physical Education, Federal University of Juiz de Fora (UFJF), Juiz de Fora, Brazil; ^7^ Department of Sports Training, Institute of Sport Sciences, The Jerzy Kukuczka Academy of Physical Education in Katowice, Katowice, Poland; ^8^ Gdansk University of Physical Education and Sport, Gdansk, Poland; ^9^ Graduate Program in Physical Education, Federal University of Rio Grande do Norte (UFRN), Natal, Brazil

**Keywords:** blood flow restriction therapy, limb arterial occlusion, cuff width, blood pressure, thigh circumference

## Abstract

**Background:** Arterial occlusion pressure (AOP) is a relevant measurement for individualized prescription of exercise with blood flow restriction (BFRE). Therefore, it is important to consider factors that may influence this measure.

**Purpose:** This study aimed to compare lower limb AOP (LL-AOP) measured with 11 cm (medium) and 18 cm (large) cuffs, in different body positions, and explore the predictors for each of the LL-AOP measurements performed. This information may be useful for future studies that seek to develop approaches to improve the standardization of pressure adopted in BFRE, including proposals for equations to estimate LL-AOP.

**Methods:** This is a cross-sectional study. Fifty-one healthy volunteers (males, *n* = 25, females, *n* = 26; Age: 18–40 years old) underwent measurement of thigh circumference (TC), brachial blood pressure, followed by assessments of LL-AOP with medium and large cuffs in positions supine, sitting and standing positions.

**Results:** The large cuff required less external pressure (mmHg) to elicit arterial occlusion in all three-body positions when compared to the medium cuff (p < 0.001). The LL-AOP was significantly lower in the supine position, regardless of the cuff used (p < 0.001). Systolic blood pressure was the main predictor of LL-AOP in the large cuff, while TC was the main predictor of LL-AOP with the medium cuff. Body position influenced strength of the LL-AOP predictors.

**Conclusion:** Our results indicate that LL-AOP and its predictors are substantially influenced by body position and cuff width. Therefore, these variables should be considered when standardizing the pressure prescribed in BFRE.

## Introduction

Blood flow restriction (BFR) training consists of performing exercise with low mechanical loads or intensities using a device capable of restricting blood flow to the exercising limb (Patterson et al., 2019). Low-load resistance training programs (20%–40% of 1-repetition maximum [1-RM]) with BFR can elicit muscle hypertrophy similar to high-load resistance training (Lixandrão et al., 2018; de Queiros et al., 2024a) with more pronounced muscle strength gains than low-load training without BFR during work-matched protocols (de Queiros et al., 2022). Therefore, this training model has been suggested as an alternative for people with limitations for high-load resistance training.

BFR can be induced by inflatable cuffs of different widths (small, 5 cm; medium, 10 cm or 12 cm; large, 17 cm or 18 cm) that are fixed to the proximal region of the exercised limb (Patterson et al., 2019). Until 2008, studies adopted arbitrary pressures to apply the BFR stimulus (Murray et al., 2021). However, this approach does not account for the individual characteristics of the participant, possibly altering the acute physiological stimulus. For example, individuals with larger limb circumferences may require more external pressure to cause arterial occlusion than those with smaller limbs (Loenneke et al., 2012). Therefore, to use a non-personalized pressure for a group of individuals does not guarantee that everyone is experiencing a similar level of BFR, potentially leading to heterogeneous BFR responses. Currently, it is recommended that pressures used in BFR training be customized based on a percentage of arterial occlusion pressure (AOP) (Patterson et al., 2019).

Cuff characteristics, body position, individual characteristics can affect AOP (de Queiros et al., 2024b). Prior research has shown supine AOP is lower than the AOP determined in the sitting and standing positions (Hughes et al., 2018; Rodrigues Neto et al., 2018; Sieljacks et al., 2018). These results can be justified by the effects of hydrostatic pressure, particularly in the lower limbs (de Queiros et al., 2024b). In this context, determining lower limb AOP (LL-AOP) in the supine position for an exercise performed in a sitting/standing position may underestimate the LL-AOP. Furthermore, the magnitude of changes in LL-AOP because of the body position adopted may vary depending on individual characteristics (e.g., anthropometric characteristics) that may generate variations in the occlusive stimulus in relation to LL-AOP.

Regarding cuff characteristics, some studies support an inverse relationship between cuff width and LL-AOP, with smaller cuffs (5 cm) requiring more external pressure to elicit arterial occlusion (Loenneke et al., 2012; Sieljacks et al., 2018; Weatherholt et al., 2019; Montoye et al., 2023). However, it is necessary to consider that most studies compare between small cuffs (5–6 cm) and medium cuffs (13–13.5 cm) therefore the results presented may not be applicable for comparisons between medium and large cuffs (≥17-cm). For example, two previous studies did not identify significant differences in LL-AOP measured with cuff widths ≥18 cm and 13-cm cuffs, suggesting the existence of a limit to the inverse relationship between LL-AOP and cuff width (Brown et al., 2018; Montoye et al., 2023).

Previous research has shown thigh circumference (TC) is the main predictor of LL- AOP for medium or small cuffs (Loenneke et al., 2012; Sieljacks et al., 2018), while brachial systolic blood pressure (SBP) was not a significant predictor. However, more recently, it was found that SBP can be a stronger predictor of LL-AOP than TC when using an 18 cm cuff to obtain the measurement (Wedig et al., 2023; Wedig et al., 2024). Furthermore, Crenshaw et al. (Crenshaw et al., 1988) identified that the correlation coefficients between LL-AOP and TC are lower in measurements taken with wider cuffs. Therefore, the evidence presented suggests that cuff width can influence the strength of LL-AOP predictors.

Currently, a limited number of studies have analyzed predictors of LL-AOP measured with 18 cm cuffs (Cirilo-Sousa et al., 2019; Wedig et al., 2024) and only one study has explored predictors of LL-AOP measured between medium and large cuffs (Wedig et al., 2023), but these studies only considered one body position. Considering the impact of body position on LL-AOP, it is relevant to analyze whether LL-AOP predictors are maintained regardless of the body position in which the measurement is performed. Given the above, this study aimed to compare LL-AOP measured with medium and large cuffs in different body positions and explore the predictors for each of the AOP measurements carried out in this study. We hypothesize that the strength of LL-AOP predictors may be influenced by cuff width and body position. Furthermore, we theorize that the difference in LL-AOP measured with large and medium cuffs is not significant, as both cuffs can interrupt the arterial pulse with similar external pressure (mmHg), while body position can promote considerable differences that may have implications for standardizing the pressure adopted in BFR exercise.

## Materials and methods

### Subjects

Fifty-one individuals (females, *n* = 26; males, *n* = 25) without known cardiovascular, metabolic and/or musculoskeletal problems, aged between 18 and 40 years, participated in this study. The sample was selected non-probabilistic way (by convenience). Participants were recruited through social media and word of mouth during January 2024. Interested participants contacted our research team and were instructed to come to our laboratory for measurements of brachial blood pressure (bBP), anthropometry and LL-AOP in supine, sitting and standing positions, with an 18 cm cuff and 11 cm. We performed a *post hoc* sample calculation to determine the power of the analyses, adopting an effect estimate of 1.5 and an α of 0.05 (t-test, difference between two dependents means), where the difference in supine LL-AOP measured with medium and large cuff was chosen as the primary outcome. As a result, we identified a power of 1.00 (Sample size = 31). G*Power software (Version 3.1.9.7) was used for these purposes.

All participants received instructions about the risks and benefits of the study and signed an informed consent form. The study was submitted and approved by the Research Ethics Committee of the local university (Opinion: 6.599.200) and was conducted following the Code of Ethics of the World Medical Association (Declaration of Helsink).

### Study design

This is a cross-sectional observational study. Initially, height and body mass were measured using a standard stadiometer and a digital scale, respectively. Subsequently, the circumference of the right thigh was measured as was done in a previous study (Loenneke et al., 2015). Participants were kept in a supine position for 10 minutes before assessment of bBP performed on the right arm. Finally, LL-AOP assessments were performed in the supine, seated, and standing positions using 11 cm and 18 cm cuffs (6 measurements). The order of LL-AOP measurements was randomized using JASP software. Subsequent LL-AOP assessments occurred after a 5-min rest between conditions to reduce hemodynamic influences of hyperemia. Evaluations were performed during the afternoon (2:00 p.m. to 4:30 p.m.) due to laboratory availability with laboratory temperature maintained at 21º–24° during the evaluations. On the day of the assessments, participants were requested not to use caffeine or perform physical exercise. There was no menstrual cycle control for female participants. This study adheres to the Strengthening the Reporting of Observational Studies in Epidemiology (STROBE) guidelines, ensuring comprehensive reporting and transparency of methods and results (Von Elm et al., 2007).

### Thigh circumference

The distance between the inguinal fold and the upper edge of the patella was measured with a steel anthropometric tape (Sanny^®^, São Paulo, Brazil) and a mark was made at the point corresponding to 33% distal to the inguinal fold. This point was chosen to measure the thigh circumference, approximate place where the cuffs would be applied similar to previous studies (Loenneke et al., 2012; Sieljacks et al., 2018).

### Brachial blood pressure

Brachial blood pressure was determined in the supine position after 10 min of rest using an automatic blood pressure measuring device (Omron^®^, HEM7200, Omron, United States). Two blood pressure measurements were taken at 1-min intervals; if a difference greater than 5 mmHg was reported between measurements, a third measurement was performed. The average value of the two closest values was adopted for analysis (Loenneke et al., 2012; Chen et al., 2021).

### Arterial occlusion pressure

**FIGURE 1:**
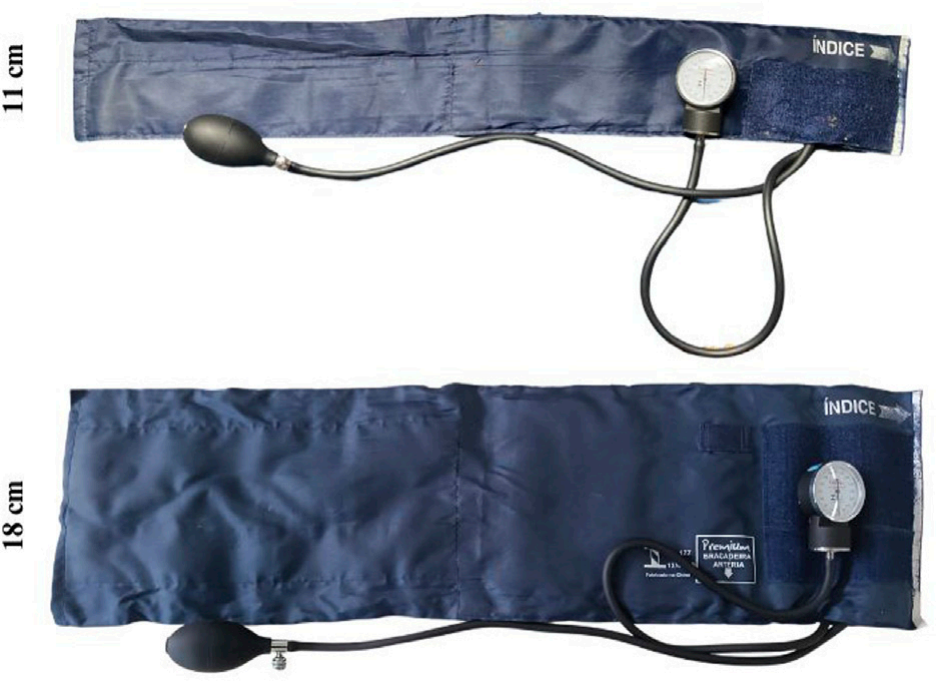
Cuffs used in the present study.

LL-AOP was determined 3-min after bBP assessments. The cuff was positioned in the proximal region of the thigh while the probe of a portable vascular Doppler (Medpej^®^, DF7001-VN; 8 MHz; Ultrasonic power: <5 mW/cm^2^) was fixed above the posterior tibial artery to identify the arterial pulse. In all assessments, the cuff bladder was positioned medially (covering the inner portion of their thigh), aiming for direct compression of the femoral artery (Spitz et al., 2020). The cuff was gradually inflated with increments of 20 mmHg until the sound signal emitted by the vascular Doppler was interrupted. Subsequently, the cuff was inflated by an additional 20 mmHg and slowly deflated to confirm the AOP values (Cirilo-Sousa et al., 2019). Participants were instructed to keep their weight evenly distributed between both legs during the assessments carried out in the standing position. To assess the LL-AOP in the seated position, participants were placed in a seated position, with their knees flexed at 90°.LL-AOP assessments were performed with a standard nylon aneroid blood pressure cuff (Premiun^®^, Brazil), with a measurement range of 0–300 mmHg and a scalar division of 2 mmHg. The cuff was 18 cm wide (18-cm x 35-cm). Therefore, a reduction in cuff width was implemented in one of our cuffs to evaluate the effect of cuff width on LL-AOP (See Figure 1).

### Statistical analyses

It was not possible to identify LL-AOP in some participants using the medium cuff, as the adapted cuff was not able to accommodate the thigh of participants with LL-AOP greater than 250 mmHg. Therefore, it was not possible use the total sample for comparison between the cuffs used in this study. The number of participants whose LL-AOP was discernible with the medium cuff varied between positions. Thus, we choose to realize isolated comparisons. The Gaussian distribution of the data was verified through the Shapiro-Wilk test (Shapiro and Wilk, 1965). Paired Student’s t-test was used to compare AOP between the medium *versus* large cuff in the three body positions. Cohen’s d was used as a measure of effect size (ES) for these comparisons. The following classification was used to interpret Cohen’s d: trivial effect (<0.19), small effect (0.20), medium effect (0.50), large effect (>0.80) (Cohen, 1992). ANOVA one way was used to analyze the effect of body position on LL-AOP. Bonferroni *post hoc* test was used to identify point differences. Independent Student’s t-test was used to compare LL-AOP determined with the large cuff between males and females.

A hierarchical linear regression model was used to determine predictors of LL-AOP measured with a medium and large cuff, in different body positions. We entered SBP first, as we formulated the possibility that this variable is highly related to LL-AOP measured with larger cuffs. Subsequently, TC and diastolic blood pressure (DBP) were entered into the model in blocks 2 and 3, respectively. The models consisted of three individual blocks to determine changes in the coefficient of determination, standard error of estimate (SEE), and the change in F value when each individual variable was added to the overall model. Multicollinearity between variables was defined as variance inflation factor (VIF) ≥ 10 and/or Pearson correlations ≥0.85. Cohen’s *f* ^*2*^ (*f* ^*2*^ = *R* ^*2*^/1-*R* ^*2*^) was calculated and presented as an effect size measure for multiple linear regressions (Espirito Santo and Daniel, 2018). Statistical significance was set *a priori* at 5%. Analyses were performed using SPSS version 24.0.

## Results

Participant characteristics are reported in Table 1. Males had significantly greater SBP, DBP, body mass index (BMI), body mass and height than females. No significant differences were reported for TC or age. The variables age, TC, BMI, SBP of the total sample are reported as median and 25th e 75th percentile, while the other variables are presented as mean and standard deviation (SD). For analyses stratified by sex, DBP, SBP and height are reported as mean and SD, and the other variables are reported as median and 25th and 75th percentile.

**TABLE 1:** Characteristics of participants.

	Total	Males	Females	p-value
Age	24 (22.0–27.0)	24 (21.5–26.5)	25 (22.75–29.0)	0.142
Height (cm)	167.9 (10.3)	175.8 (7.4)	160.4 (6.4)	<0.001
Body mass (kg)	68.09 (13.4)	83 (65.25–86.6)	59.7 (53.1–66.62)	<0.001
BMI (kg/m^2^)	24.6 (21.38–26.4)	25.4 (22.44–27.44)	23.01 (20.93–25.65)	0.014
Circumference thigh (cm)	59.2 (52.6–61.0)	59.7 (52.1–63.0)	57.9 (53.2–59.7)	0.070
SBP (mmHg)	113 (108.5–123.5)	120.14 (11.7)	110.8 (7.6)	0.002
DBP (mmHg)	62.8 (8.01)	59 (7.4)	66 (6.7)	<0.001

Note: BMI, body mass index; DBP, diastolic blood pressure; SBP, systolic blood pressure.

It was possible to measure the LL-AOP in the three positions of all participants using the large cuff. However, it was not possible to measure the LL-AOP of all participants using the medium cuff (11 cm); in the supine, sitting and standing positions, it was not possible to measure the LL-AOP of 19, 18 and 25 participants, respectively.

### Cuff size and body position

**FIGURE 2:**
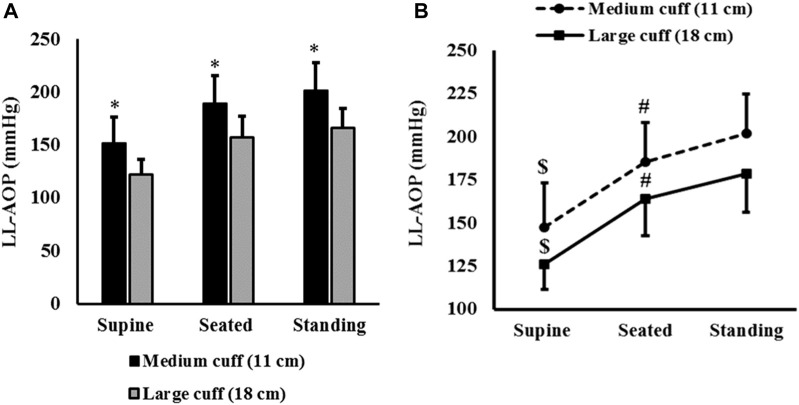
LL-AOP (mean and SD) in different body positions. Note: * = significant difference between cuffs (p < 0.001); $ = significantly lower than LL-AOP measured in seated and standing positions (p < 0.001); # = significantly lower than LL-AOP measured in standing positions (p < 0.001).

Significant differences were reported in LL-AOP between medium and large cuffs in the supine position (t _(31)_ = 8.954; ∆ = 29.6 mmHg; confidence interval 95% [CI95%] = 22.9, 36.4; p < 0.001; d = 1.5; Figure 2A), seated position (t _(30)_ = 7.468; ∆ = 32.7 mmHg; CI95% = 23.8, 41.7; p < 0.001; d = 1.3; Figure 2A) and standing position (t (26) = 8.3; ∆ = 35.8 mmHg; CI95% = 27.0, 44.6; p < 0.001; d = 1.6; Figure 2A).

ANOVA revealed an effect of body position on LL-AOP measured by the medium cuff (F_(2,52)_ = 140.223; p < 0.001; η^2^p = 0.844) and large cuff (F_(2,100)_ = 287.557; p < 0.001; η^2^p = 0.852). For both cuffs, LL-AOP measured in the supine position was lower than LL-AOP measured in seated position (p < 0.001) and standing (p < 0.001); significant differences were also reported between seated and standing position (p < 0.001). The average LL-AOP presented in the three body positions is reported in Figure 2B.

### Sex differences

**FIGURE 3:**
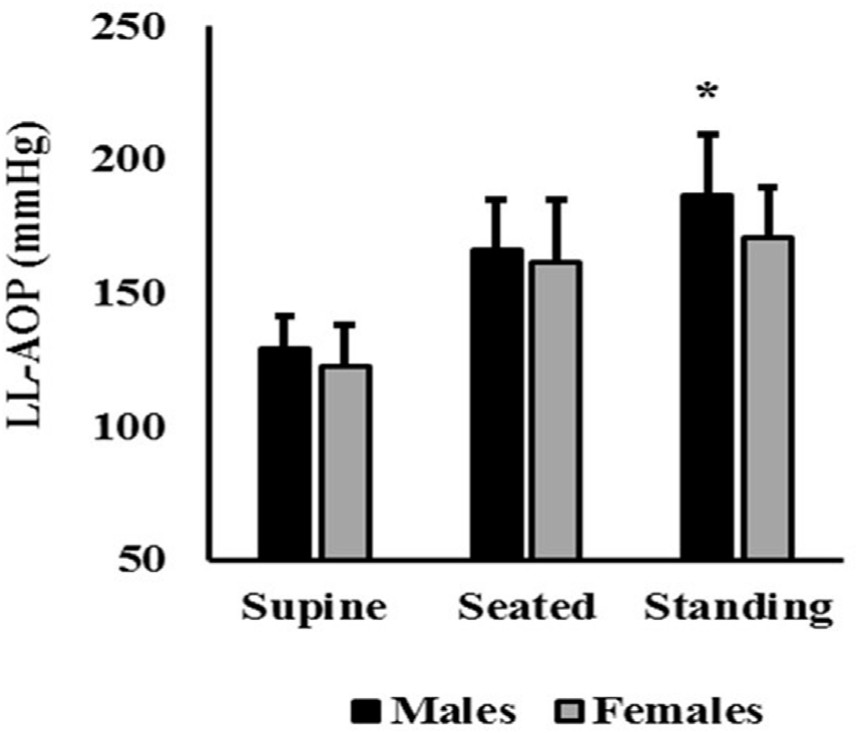
LL-AOP (mean and SD) comparisons between males and females. Note: * = significant difference between means (p = 0.01).

No significant differences between sexes and LL-AOP was observed for the supine position (t_(49)_ = 1.759; ∆ = 7 mmHg; CI95% = −0.9, 14.9; p = 0.085) and seated position (t_(49)_ = 0.771; ∆ = 4.6 mmHg; CI95% = −7.4, 16.6; p = 0.44), but males presented higher values than females in standing position (t_(49)_ = 2.669; ∆ = 15.7 mmHg; CI95% = 3.8, 27.5; p = 0.010; d = 0.8). Furthermore, the difference in LL-AOP between seated and standing positions (20.5 mmHg *versus* 9.4 mmHg for male and female, respectively; p = 0.023) and between supine and standing (57.1 mmHg *versus* 48.3 mmHg for males and females; respectively; p = 0.044) was significantly higher in males. The average LL-AOP presented in the three body positions segmented by sex is reported in Figure 3.

### Determinants of lower body arterial occlusion pressure

Hierarchical regression models for the large cuff are found in Table 2. For model 1, SBP independently explained approximately 35% of the variance in supine LL-AOP. Entering TC in block 2 did not explain additional variance (Sig. F change = 0.100). A similar result was identified in block 3 which included SBP, TC and DBP (Sig. F change = 0.084).

**TABLE 2:** Multiple regression analysis of variables influencing arterial occlusion pressure (18 cm cuff).

Variable	Stand. β	p	*R* ^*2*^ (*f* ^*2*^)	Adj. *R* ^*2*^	Sig. F Change	SEE	Mean square error
18-cm cuff (supine)							
Block 1			0.358	0.345	<000.1	11.704	136.9
SBP	0.599	<0.001					
Block 2			0.394 (0.65)	0.369	0.100	11.493	132.0
SBP	0.526	<0.001					
33% Circumference	0.202	0.100					
Block 3			0.431 (0.75)	0.395	0.084	11.248	126.5
SBP	0.456	0.001					
33% Circumference	0.253	0.043					
DBP	0.207	0.084					
18-cm cuff (seated)							
Block 1			0.303	0.289	<000.1	17.972	322.9
SBP	0.550	<0.001					
Block 2			0.373 (0.59)	0.346	0.025	17.227	296.7
SBP	0.449	0.001					
33% Circumference	0.283	0.025					
Block 3			0.401 (0.66)	0.363	0.142	17.012	289.3
SBP	0.388	0.004					
33% Circumference	0.327	0.012					
DBP	0.179	0.142					
18-cm cuff (standing)							
Block 1			0.397	0.385	<0.001	17.473	305.3
SBP	0.630	<0.001					
Block 2			0.566 (1.30)	0.548	<0.001	14.981	224.4
SBP	0.472	<0.001					
33% Circumference	0.440	<0.001					
Block 3			0.568 (1.31)	0.540	0.640	15.104	228.1
SBP	0.489	<0.001					
33% Circumference	0.428	<0.001					
DBP	−0.048	0.640					

Note: DBP, diastolic blood pressure; SBP, systolic blood pressure; SEE, standard error of estimate.

In model 2, SBP independently explained approximately 29% of the variance in seated LL-AOP. Entering TC in block 2 explained additional variance of approximately 6%. Standardized betas indicated that SBP explained the greatest variance. The entry of DBP in block 3 did not explain additional variance (Sig. F change = 0.142).

In model 3, SBP independently explained approximately 38.5% of the variance in standing LL-AOP. Entering TC in block 2 explained additional variance of approximately 16.5%. Standardized betas indicated that SBP explained the greatest variance. The entry of DBP in block 3 did not explain any additional variance (Sig. F change = 0.640).

The hierarchical regression models for the medium cuff are found in Table 3. Block 3 of model 1, composed of SBP, TC and DBP, explained the greatest variance in supine LL-AOP; however, SBP was not a significant predictor of the model. Standardized betas indicated that TC explained the greatest variance in blocks 2 and 3.

**TABLE 3:** Multiple regression analysis of variables influencing arterial occlusions pressure (11 cm cuff).

Variable	Stand. β	p	*R* ^*2*^ (*f* ^*2*^)	Adj. *R* ^*2*^	Sig. F Change	SEE	Mean square error
11-cm cuff (supine)							
Block 1			0.213	0.187	0.008	22.507	506.5
SBP	0.462	0.008					
Block 2			0.430 (0.75)	0.390	0.002	19.489	379.8
SBP	0.360	0.018					
33% Circumference	0.476	0.002					
Block 3			0.526 (1.1)	0.475	0.024	18.089	327.2
SBP	0.262	0.071					
33% Circumference	0.519	0.001					
DBP	0.325	0.024					
11-cm cuff (seated)							
Block 1			0.136	0.106	0.041	24.175	584.4
SBP	0.368	0.041					
Block 2			0.631 (1.71)	0.604	>0.001	16.084	258.6
SBP	0.185	0.131					
33% Circumference	0.727	>0.001					
Block 3			0.653 (1.88)	0.614	0.203	15.886	252.3
SBP	0.143	0.249					
33% Circumference	0.739	>0.001					
DBP	0.153	0.153					
11-cm cuff (standing)							
Block 1			0.253	0.223	0.007	22.750	517.5
SBP	0.503	0.007					
Block 2			0.592 (1.42)	0.558	>0.001	17.167	294.7
SBP	0.338	0.020					
33% Circumference	0.665	>0.001					
Block 3			0.594 (1.46)	0.541	0.734	17.491	305.9
SBP	0.319	0.042					
33% Circumference	0.611	>0.001					
DBP	0.049	0.734					

Note: DBP, diastolic blood pressure; SBP, systolic blood pressure; SEE, standard error of estimate.

Block 2 of model 2 composed of SBP and TC explained 60.4% of the variance in seated LL-AOP; however, SBP was not a significant predictor. The entry of DBP in block 3 did not explain the additional variance (Sig. F change = 0.203). Similarly, block 2 of model 3, composed of SBP and TC, explained the greatest variance in standing LL-AOP; standardized betas indicated that TC explained the most variance in block 2. Entering DBP in block 3 did not explain any additional variance (Sig. F change = 0.734).

## Discussion

This study compared LL-AOP measured with medium and large cuffs in different body positions and explored predictors for each of the LL-AOP measurements performed in this study. It was possible to identify an inverse relationship between LL-AOP and cuff width, with the medium cuff requiring more external pressure to elicit arterial occlusion regardless of the body position adopted for measurement. We identified that body position significantly affects LL-AOP, with higher mean LL-AOP values in measurements taken in the standing position. Regarding the determinants of lower limb LL-AOP, SBP was the main determinant with the large cuff, while TC was the main determinant of LL-AOP measured with the medium cuff.

### Cuff width and lower limb arterial occlusion pressure

The effect of cuff width on AOP has been explored in some studies that, in most cases, were comparisons between small cuffs and medium cuffs (Loenneke et al., 2012; Sieljacks et al., 2018; Weatherholt et al., 2019; Montoye et al., 2023). The results from these studies indicate that AOP is significantly higher when measured using small cuffs, indicating an inverse relationship between cuff width and AOP. Our results support this relationship and extend it to larger cuffs, since the mean LL-AOP obtained with the large cuff was significantly less than the mean LL-AOP obtained with a medium cuff, regardless of the body position in which the measurement was performed. However, when comparing the magnitude of the difference reported in the studies, it is possible to identify a certain level of heterogeneity. For example, the mean absolute difference reported by Sieljacks et al. (Sieljacks et al., 2018) in the supine LL-AOP with a medium (13 cm) and a small (6 cm) cuff was 200 mmHg (ES: 2.2), while the difference reported in the present study between the LL-AOP measured with a medium and a large cuff was only 29.68 mmHg (ES: 1.5).

The heterogeneity in the magnitude of differences in LL-AOP reported in our study and in the study by Sieljacks et al. (Sieljacks et al., 2018) can be justified by the fact that we performed comparisons between medium cuffs *versus* large cuffs, while the Sieljacks et al. (Sieljacks et al., 2018) carried out comparisons between medium cuffs and small cuffs. This is supported by the results presented by Montoye et al. (Montoye et al., 2023); when comparing the LL-AOP measured with a medium cuff (11.5 cm) and a large cuff (21 cm), the authors identified an absolute mean difference of 32.6 mmHg (ES: 1.29), results similar to this study. In conjunct, the evidence presented supports that the magnitude of differences in LL-AOP is more pronounced in comparisons made between medium and small cuffs, relative to comparisons made between medium and large cuffs.

Although the magnitude of the differences reported in our study is relatively small, it is still present, diverging from some studies that made comparisons between medium and large cuffs that did not identify significant differences (Brown et al., 2018; Montoye et al., 2023). Unlike the current study, the other authors compared a 13 cm cuff *versus* 18 cm and 21 cm cuffs. We speculate that the inverse relationship between LL-AOP and cuff width is limited to cuff widths less than 13 cm width.

The differences presented in the AOP due to the cuff width must be considered by professionals implementing BFR. For example, selecting arbitrary pressures based on previous studies while disregarding the impact cuff width can be problematic. Applying a certain arbitrary pressure to a small cuff is unlikely to be the same applied stimulus when applying the same pressure to a cuff with a different width. In support, a previous study identified that applying pressure intended for a 5 cm cuff (40% of AOP determined with a 5 cm cuff, 142 ± 26 mmHg) to a 12 cm cuff resulted in a higher perceived discomfort than the exercise performed with the correct pressure (40% of AOP determined with a 12 cm cuff, 60 ± 9 mmHg), in addition to a lower number of repetitions (Spitz et al., 2021). We must point out that we do not know whether a similar result would be reported if a similar experiment were carried out with the cuffs adopted in the current study, since the differences in LL-AOP measured with a medium and large cuff were relatively small. To illustrate, 60.7 ± 9.8 mmHg and 50.9 ± 6.9 mmHg were the average values corresponding to 40% of the supine LL-AOP obtained with the medium and large cuff, respectively. This small difference may not impact BFR exercise.

Previously, it was found that smaller cuffs generate greater discomfort than larger cuffs inflated to the same relative pressure (%AOP) (Estebe et al., 2000; Spitz et al., 2021). Possibly, these results can be justified by the fact that a higher external pressure (mmHg) is necessary to interrupt the arterial pulse when smaller cuffs are used to measure AOP. In this sense, cuff width can be considered an important variable in selecting the most comfortable cuff for BFR training.

Multiple linear regression models were used to identify predictor variables of LL-AOP determined with cuffs of different widths in different body positions. Based on previous literature, the models tested were composed of three variables, SBP, TC and DBP (Loenneke et al., 2012; Sieljacks et al., 2018). For the 18 cm cuff, SBP was the main predictor of LL-AOP, regardless of body position, although the magnitude of the standardized coefficient may be influenced by body position. Our results are in line with a recent publication (Wedig et al., 2024). Wedig et al. (Wedig et al., 2024) identified that SBP was the strongest predictor of LL-AOP measured with an 18 cm cuff in the sitting position, in a model involving TC, DBP and sociodemographic variables. Taken together, the results presented provide evidence that SBP may be the strongest predictor of LL-AOP determined with cuff widths ≥18 cm.

In contrast, for 11 cm cuff, TC was the main predictor of LL-AOP regardless of body position. Therefore, the influence of TC is reduced as the cuff width increases. These results are in line with the results presented by Crenshaw et al. (Crenshaw et al., 1988). These authors identified that Pearson’s correlation coefficients between thigh circumference and LL-AOP were lower for larger cuffs compared to smaller cuffs (*r* = 0.89, *r* = 0.77 and *r* = 0.44 for 4.5 cm, 12 cm, and 18 cm cuffs, respectively). Possibly, the reduced influence of TC on LL-AOP as cuff width increases can be explained by the fact that wider cuffs transmit pressure more efficiently to the underlying soft tissues (Crenshaw et al., 1988). In turn, the lesser influence of TC on LL-AOP may increase the influence of hemodynamic variables on LL-AOP.

Considering that the LL-AOP predictor variables can be substantially influenced by the cuff width, equations designed to estimate LL-AOP with smaller cuffs (e.g., 5 cm and 12 cm) (Sieljacks et al., 2018) are certainly not valid for estimating LL-AOP with large cuffs (≥18 cm). This aspect should be considered by professionals looking for options to estimate the LL-AOP in the absence of doppler ultrasound assessment or other approaches.

### Body position and lower limb arterial occlusion pressure

It was possible to identify that LL-AOP is significantly decreased in measurements taken in the supine position when compared to measurements taken in sitting and standing positions. These results are in line with previous studies (Hughes et al., 2018; Sieljacks et al., 2018) and can be explained by the impact of hydrostatic pressure. We observed a moderate and significant positive correlation (*r* = 0.42; p < 0.05) between height and LL-AOP measured in the standing position, but not in the supine or sitting position regardless of the cuff used. Furthermore, the effects of hydrostatic pressure could support the reported sex difference in LL-AOP measured in the standing position using the large cuff, considering that the sex difference was only found in the standing position and the average height of the male volunteers was significantly higher than the average height of the female volunteers.

The mean difference between LL-AOP measured in the supine and standing positions was 52.6 and 54.5 mmHg for medium and large cuffs, respectively, but the magnitude of the difference varied between 6 and 88 mmHg for the large cuff and between 0 and 92 mmHg for the medium cuff. The individual differences reported in our sample can explain this substantial variation. To explore a possible influence of height on the magnitude of differences in LL-AOP as a function of body position, we implemented bivariate correlations between height and the difference of LL-AOP measured in the standing position and the supine and sitting position. We identified a moderate positive correlation between height and difference between LL-AOP measured in the standing and supine position for the wide cuff (*n* = 51; *r* = 0.46; p = 0.001) and the medium cuff (*n* = 27; *r* = 0.456; p = 0.018). Furthermore, TC was a stronger predictor of AOP in measurements taken in standing and sitting positions relative to LL-AOP measured in the supine position. Given that individual characteristics can influence the magnitude of changes in AOP depending on body position, it is possible that there are variations in the stimulus imposed when customizing BFR training pressure based on supine LL-AOP for an exercise performed in a standing position, although exercise trials should be done to confirm this assertion.

This study has certain limitations that need to be highlighted. First, it was not possible to blind the investigators. Secondly, our data are specific to 11 cm and 18 cm cuffs, so they should not be extrapolated to cuffs with other widths. Finally, our results are unique to young, healthy adults.

## Conclusion

In conclusion, the present study shows that an 18 cm cuff requires less external pressure to elicit arterial occlusion than an 11 cm cuff, although the difference was relatively small. In addition, this study demonstrated that body positions significantly affect LL-AOP, with higher mean LL-AOP values in measurements performed in the standing position. For standardizing BFR pressure, it is important that the body position adopted during exercise must be considered when measuring LL-AOP. In terms of determination of the LL-AOP, SBP was the main determinant with the large cuff while TC was the main determinant of LL-AOP measured with the medium cuff. Therefore, an equation developed to estimate the LL-AOP determined with a medium cuff is not valid for estimating the LL-AOP determined with a large cuff. Similarly, an equation developed to estimate supine LL-AOP may not be valid for estimating LL-AOP determined in the standing position.

## Data Availability

The raw data supporting the conclusions of this article will be made available by the authors, without undue reservation.
